# Inflamed adipose tissue: A culprit underlying obesity and heart failure with preserved ejection fraction

**DOI:** 10.3389/fimmu.2022.947147

**Published:** 2022-11-22

**Authors:** Chenyu Li, Donglu Qin, Jiarui Hu, Yang Yang, Die Hu, Bilian Yu

**Affiliations:** ^1^ Department of Cardiovascular Medicine, the Second Xiangya Hospital, Research Institute of Blood Lipid and Atherosclerosis, Central South University, Changsha, Hunan, China; ^2^ Department of Spine Surgery, the Second Xiangya Hospital, Central South University, Changsha, Hunan, China

**Keywords:** obesity, HFPEF, inflamed adipose tissue, NLRP3 inflammasome, inflammation

## Abstract

The incidence of heart failure with preserved ejection fraction is increasing in patients with obesity, diabetes, hypertension, and in the aging population. However, there is a lack of adequate clinical treatment. Patients with obesity-related heart failure with preserved ejection fraction display unique pathophysiological and phenotypic characteristics, suggesting that obesity could be one of its specific phenotypes. There has been an increasing recognition that overnutrition in obesity causes adipose tissue expansion and local and systemic inflammation, which consequently exacerbates cardiac remodeling and leads to the development of obese heart failure with preserved ejection fraction. Furthermore, overnutrition leads to cellular metabolic reprogramming and activates inflammatory signaling cascades in various cardiac cells, thereby promoting maladaptive cardiac remodeling. Growing evidence indicates that the innate immune response pathway from the NLRP3 inflammasome, to interleukin-1 to interleukin-6, is involved in the generation of obesity-related systemic inflammation and heart failure with preserved ejection fraction. This review established the existence of obese heart failure with preserved ejection fraction based on structural and functional changes, elaborated the inflammation mechanisms of obese heart failure with preserved ejection fraction, proposed that NLRP3 inflammasome activation may play an important role in adiposity-induced inflammation, and summarized the potential therapeutic approaches.

## Introduction

The incidence of heart failure (HF) is rapidly increasing. As of 2017, an estimated 64.3 million individuals have been diagnosed with HF worldwide (1). More than half of the patients with HF have HF with preserved ejection fraction (HFpEF), and the incidence rate of HFpEF is still increasing (2). HFpEF, which is characterized by an ejection fraction (EF) >50% with diastolic dysfunction, high filling pressures, and exercise intolerance (3), is a heterogeneous syndrome that exhibits different phenotypes due to various risk factors, such as obesity, hypertension, and diabetes.

The global number of people with obesity is increasing at an astonishing rate. By 2020, the populations of overweight and obesity worldwide have reached 1.9 billion and 650 million, respectively (4). Obesity is a prevalent and independent risk factor for HF, regardless of the ejection fraction. Many studies have demonstrated that patients with obesity have a greater risk of HFpEF than of HF with reduced ejection fraction (HFrEF), and excess weight and obesity are also prevalent in patients with HFpEF (83%) (5, 6). Compared to other types of HFpEF, obese HFpEF has unique hemodynamic and structural abnormalities, in which chronic inflammatory conditions play a crucial role in its emergence and development (7, 8). Therefore, this review identifies inflammation as a specific etiology of obese HFpEF, describes the pathophysiological mechanisms of inflammation in obese HFpEF, and proposes potential anti-inflammatory therapies.

## Evidence supporting the existence of obese HFpEF

### Anatomical structure and histological changes of the heart in obesity

Early autopsy reports have found increased heart weight and ventricular hypertrophy in patients with obesity, more commonly with left ventricular (LV) hypertrophy than the right (9, 10). Animal studies have also shown that the LV wall thickens, and its volume increases in the early stages of obesity (11). Therefore, the cardiac structural changes caused by obesity are mainly LV remodeling, with increased wall thickness more than dilatation. Two forms of LV hypertrophy, concentric and eccentric, have been found in individuals with obesity; however, concentric hypertrophy is more common (12, 13). It is noting that the different geometries of ventricular hypertrophy in individuals with obesity may be associated with different insulin sensitivity states and not all obese individuals develop LV hypertrophy. Sciacqua et al. (14) found that ventricular remodeling in metabolically unhealthy obese individuals showed more eccentric hypertrophy than that in metabolically healthy individuals, implying that obesity-related metabolic abnormalities play a role in ventricular hypertrophy. Another cross-sectional study also suggested that metabolic abnormalities, but not metabolically healthy obesity, were significantly associated with LV hypertrophy (15).

Although relatively rare, right ventricular (RV) thickness and volume can also increase in individuals with obesity. Preliminary studies have suggested that RV hypertrophy and dilatation are secondary to pulmonary hypertension due to obesity-induced LV failure, sleep apnea, and hypoventilation (16, 17). However, subsequent research (18) has demonstrated that body mass index (BMI) is the most relevant clinical factor for biventricular hypertrophy in individuals with obesity without pulmonary hypertension. Wong et al. (19) also found that increased BMI in subjects with overweight and obesity was associated with severity of RV dysfunction, independent of sleep apnea. These data suggested a direct correlation between obesity and RV hypertrophy.

In addition to macro-anatomical structures, the hearts of patients with obesity often undergo multiple morphological changes at the cellular level. Diffuse cardiomyocyte hypertrophy is the most specific histological feature of obesity-associated cardiac injury. Early clinical research found that compared with patients without obesity but with HF, cardiomyocyte hypertrophy is more evident in patients with obesity and HF (20). Despite cardiomyocyte remodeling, histological changes are different in concentric and eccentric hypertrophy. In contrast to concentric hypertrophy, in which cardiomyocytes grow only laterally, in eccentric hypertrophy, cardiomyocytes grow proportionally in both longitudinal and lateral directions (21).

Both animal and human studies have demonstrated microvascular rarefaction, interstitial fibrosis, and cellular infiltration in the myocardium of patients with obesity-related HFpEF (22, 23). Myocardial microvascular density is decreased in obesity-related HFpEF, which causes cardiac hypoperfusion owing to reduced myocardial oxygen delivery (24, 25). Furthermore, increased type I collagen and transforming growth factor (TGF)-β1 levels proved the interstitial fibrosis increase in the hearts of obese animal models (22, 26). Interstitial fibrosis mainly refers to the complex process of excessive proliferation of myocardial interstitial fibroblasts, abnormal distribution, and excessive deposition of collagen caused by various stresses, such as chronic hypoxia and increased oxidative stress (26, 27). Studies have also found that the expression of the pro-inflammatory macrophage markers monocyte chemoattractant protein-1 (MCP-1) and CD11c increased in the myocardial mRNA of obese mice, suggesting that obesity induces inflammatory cells and factors infiltration of cardiomyocytes (22), implying the role of inflammation in HFpEF.

### Cardiac functional consequences associated with obesity

Studies have shown that LV diastolic dysfunction, rather than systolic dysfunction, represents the earliest sign of cardiac involvement in patients with obesity (28, 29). Diastolic dysfunction may precede systolic dysfunction or may occur simultaneously with systolic dysfunction. The typical diastolic dysfunction in patients with obesity is abnormal diastolic filling or decreased myocardial compliance, which manifests as higher left ventricular end-diastolic pressure (LVEDP) and lower mean mitral valve E/A ratio on echocardiography (28, 30). In addition, a load-dependent myocardial tissue imaging technique revealed increased myocardial stiffness in patients with obesity, further confirming abnormal myocardial relaxation (31, 32). However, there are inconsistent findings regarding the effect of obesity on LV systolic function in early studies. Several studies have shown no obvious difference in the mean LV systolic fraction between subjects with moderate to severe obesity and lean subjects (33, 34). In contrast, many studies have found that subjects with moderate obesity had a remarkably lower mean left ventricular ejection fraction (LVEF) than lean subjects (35, 36). Notably, despite the decline, the median LV systolic function in these studies was always within the normal range for obesity (33–36). Thus, the negative results in certain studies may be due to the load dependence and insensitivity of the criteria employed to assess the LV contractile function.

In recent years, methods to assess systolic function by quantifying myocardial strain have been proposed, including echocardiography to measure circumferential fiber-shortening velocity, tissue Doppler echocardiography, cardiac magnetic resonance tissue labeling, and feature tracking (37–39). Precise measurements using more sensitive approaches showed that the myocardial tissue strain index and velocity decrease proportionally with increasing obesity, even if the LVEF remains normal, suggesting that obesity is implicated in subclinical systolic dysfunction (40, 41). Obokata et al. (42) also found that, while LVEF was similar in both the obese HFpEF group and controls, LV systolic function was impaired in patients with obese HFpEF, as evidenced by decreased longitudinal strain.

Obesity not only leads to LV dysfunction but also RV dysfunction. Studies have found that the RV stroke volume and RV ejection fraction are lower in individuals with overweight and obesity, even if the LV parameters are adjusted, supporting the opinion that RV dysfunction in HFpEF may be parallel to LV dysfunction rather than a consequence of HF deterioration (42, 43). Thomas et al. (44) and Aschaueret et al. (45) confirmed that 19%–25% of patients with HFpEF present with RV dysfunction. Furthermore, RV dysfunction in HFpEF in these studies was primarily determined at rest. Recent studies have indicated that RV reserve is impaired during exercise in patients with HFpEF, even though RV systolic and diastolic functions may remain normal at rest (46), implying a higher incidence of RV dysfunction.

### Difference between HFpEF with and without obesity

Compared with patients with HFpEF without obesity, patients with obese HFpEF have significantly different hemodynamics and a unique pattern of myocardial remodeling. At rest, patients with obese HFpEF show higher right atrium (RA) pressure and pulmonary capillary wedge pressure (PCWP). During exercise, obese HFpEF patients had higher left and right heart filling pressures and higher pulmonary artery pressures which induced more severe RV dysfunction. In addition to RV dysfunction, increased left ventricular mass and volume, and the increased ratio of the two indicated greater concentric remodeling in obese HFpEF (32, 42). Profound plasma volume overload, increased pericardial restraint caused by increased epicardial fat thickness, and enhanced ventricular interaction are contributors to the unique hemodynamic derangements and ventricular remodeling in HFpEF patients with obesity.

Despite more severe symptoms and hemodynamic abnormalities, brain natriuretic peptide (BNP) levels, which are a critical diagnostic tool for HF, are lower in patients with obesity than those without obesity. Multiple studies have found an inverse correlation between BMI and BNP levels (47, 48). Tarama et al. (49) further found that BNP levels were relatively lower in HF patients with overweight and obesity. In contrast, another study found that BNP and N-terminal pro-B-type natriuretic peptide (NT-proBNP) concentrations increase after weight loss in patients with obesity (50). Enhanced BNP degradation in the adipose tissue upon the action of adipocyte-derived neprilysin may contribute to the spuriously low BNP levels observed in patients with HF (51). In addition, the changes of signal natriuretic peptide receptor (NPR) - A and clearance of NPR-C receptor in adipose tissue may also be one of the reasons (52).

Low BNP levels in the obese HFpEF phenotype indicate that measurement of natriuretic peptides may be misleading in its diagnosis. Therefore, it is necessary to explore other specific biomarkers of the obese phenotype. Obese HFpEF has been associated with higher levels of inflammatory markers, such as interleukin-6 (IL-6), tumor necrosis factor-α (TNF-α), and soluble growth stimulation expressed in gene 2 (53, 54). Based on differences in plasma biomarkers, response to spironolactone, echocardiography, and arterial tone, HFpEF has been classified into three types in the literature (55). Among them, phenotype group 3, prevalent in patients with obesity and diabetes mellitus, was characterized by systemic inflammation with elevated levels of inflammatory factors, especially TNF-α and renin, and a preferential response to spironolactone (55, 56). These results demonstrate that obese HFpEF differs from other types of HFpEF, and this difference suggests distinct underlying mechanisms across the clinically identifiable phenol groups of HFpEF.

## Unique mechanisms of obese HFpEF

**Figure 1:**
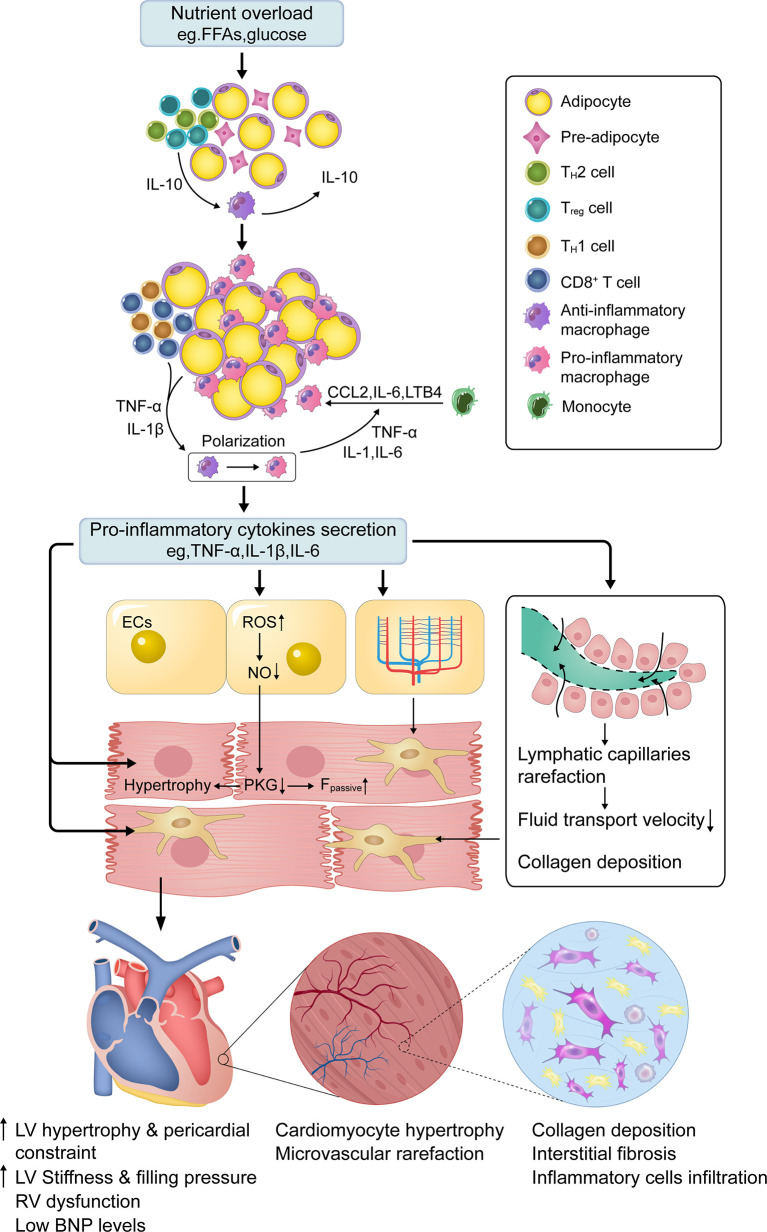
Central role of low-grade systemic inflammation in obese HFpEF. In normal adipose tissue, T_H_2 and T_reg_ polarized T cells predominate in ATTs. These T cells secrete IL-10, which stimulates resident ATMs to secrete IL-10 to limit inflammation. Chronic nutrient overload induces adipose tissue expansion and a shift in ATTs such that CD8^+^ and T_H_1 polarized T cells dominate, which recruit circulating monocytes to adipose tissue through chemokines (e.g., CCL2). Under the stimulation of cytokines (e.g., TNF-α and IL-1β) secreted by ATTs and enlarged adipocytes, ATMs polarize towards a pro-inflammatory macrophage phenotype and attract additional monocytes by secreting pro-inflammatory cytokines (e.g., IL-6, IL-1, and TNF-α). This forms a vicious cycle in which adipose tissue becomes inflamed. High levels of circulating proinflammatory cytokines activate inflammatory cascades in different cardiac cells, which are associated with cellular dysfunction. Among them, coronary microvascular endothelial ROS overproduction and dysfunction drive cardiomyocyte hypertrophy and stiffening, as well as interstitial fibrosis, by lowering myocardial NO bioavailability and PKG activity, which is the critical mechanism for obese HFpEF. Microvascular and lymphatic capillary rarefaction also contributes to interstitial fibrosis through insufficient cardiac perfusion and poor lymphatic transport. *FFAs—free fatty acids; ATTs—adipose tissue T cells; ATMs—adipose tissue macrophages; IL-1/1β/6/10/18—interleukin-1/1β/6/10/18; CCL2—C-C motif chemokine ligand 2; LTB4—leukotriene B4; TNF-α—Tumor necrosis factor-alpha; ECs—endothelial cells; NO—nitric oxide; PKG—protein kinase G; ROS—reactive oxygen species; F_passive_—cardiomyocyte resting tension; RV—right ventricle; IVS—interventricular septum; LV—left ventricle; HFpEF—heart failure with preserved ejection fraction; BNP*, *Brain Natriuretic Peptide*.

**Figure 2:**
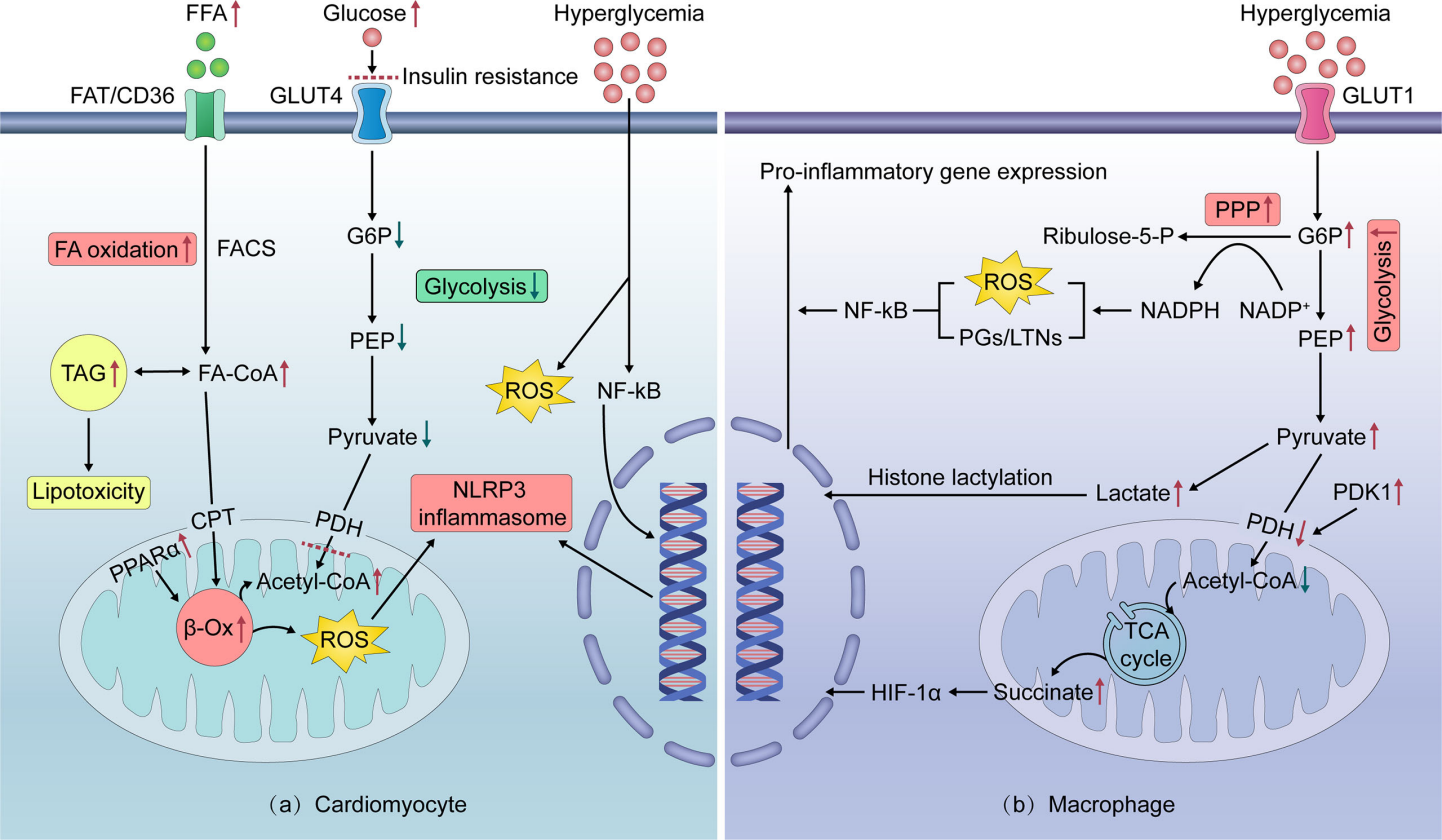
Intracellular mechanisms of cardiomyocyte and pro-inflammatory macrophage metabolic reprogramming in obesity. **(A)** In nutrient overloaded cardiomyocytes, excessive FFA intake activates PPARα, thereby enhancing FA β-oxidation. This leads to excess ROS production, which activates the NLRP3 inflammasome to promote the secretion of inflammatory cytokines. Excess FA is stored as TAGs, which can mediate lipotoxicity. Increased FA oxidation contributes to cardiomyocyte IR by inhibiting GLUT4-mediated glucose uptake and utilization, resulting in cardiomyocyte exposure to high glucose levels. Hyperglycemia can induce ROS overproduction and directly trigger a series of pro-inflammatory pathways in cardiomyocytes. **(B)** In nutrient overloaded macrophages, glucose intake *via* insulin-independent GLUT1 and metabolism reprogram to rapid aerobic glycolysis. The product of glycolysis, lactate, increases histone lactation, thereby inducing the pro-inflammatory polarization of macrophages. Glycolysis also generates NADPH through the PPP, which promotes the production of ROS and supports proinflammatory prostaglandin/leukotriene synthesis, thereby activating NF-kB. In addition, enhanced glycolysis induces a truncated tricarboxylic acid cycle and succinate accumulation *via* decreased pyruvate entry into the mitochondria, which promotes HIF-1α activation and pro-inflammatory gene expression. *FA—fatty acid; FFAs—free fatty acids; FAD/CD36—fatty acid translocase; TAG*—*triacylglycerol; FACS—fatty acyl-coenzyme A synthetase; FA-CoA*—*fatty acyl-coenzyme A; CPT—carnitine palmitoyltransferase; PDH—pyruvate dehydrogenase complex; PDK1—pyruvate dehydrogenase kinase 1; Acetyl-CoA—Acetyl coenzyme A; PPARa—peroxisome proliferator-activated receptor alpha; β-Ox—beta-oxidation; G6P*—*glucose-6-phosphate; PEP—phosphoenolpyruvate; PPP—pentose phosphate pathway; NADPH—nicotinamide adenine dinucleotide phosphate; NADP+—oxidized form of nicotinamide adenine dinucleotide phosphate; PGs—prostaglandins; LTs/LTNs—leukotrienes; TCA cycle—tricarboxylic acid cycle; HIF-1α—hypoxia-inducible factor-1 alpha; Ribulose-5-P—ribulose-5-phosphate; PPARα—peroxisome proliferator-activated receptor α; ROS—reactive oxygen species; NLRP3—NOD-like receptor family, pyrin domain containing 3; TAGs—triacylglycerols; IR—insulin resistance; GLUT1/4—glucose transporters 1/4; IL-1β/6/12—interleukin-1β/6/12; TNF-α—Tumor necrosis factor-αlpha; NF-κB—nuclear factor kappa B*.

Various central pathophysiological anomalies have shown indications of being associated with the obese HFpEF phenotype, such as energy substrate metabolism changes, neurohormonal perturbations, and sodium retention (57). Inflammation has been extensively studied in recent years as a significant pathophysiological change in obese heart failure (54). Chronic nutrient overload, as the initiating factor of obesity, promotes adipose tissue expansion and activates inflammatory responses with the overproduction of pro-inflammatory cytokines and subsequent systemic inflammation and cardiac remodeling (58, 59) (**Figure 1**); on the other hand, overnutrition contributes to cellular metabolic reprogramming and exerts direct pro-inflammatory effects on different kinds of cardiac cells, which is also an important reason for HFpEF (59, 60) (**Figure 2**).

### Inflamed adipose tissue and low-grade systemic inflammation in obesity

Excessive intake of nutrients by the body increases insulin secretion and induces the transport and storage of adipose and muscle cells in the form of triglycerides and glycogen (61). Adipose tissue is a multifaceted fat depot and a cellular energy sensor. Adipocytes autonomously sense energy stores; accordingly, they initiate a feedback circuit by secreting adipokines that reduce food intake and activate the sympathetic nervous system to increase thermogenesis and lipolysis (62, 63). In a state of continuous positive energy balance, the constant deposition of triglycerides results in an increase in the size and number of adipocytes. However, these cellular adaptive changes will reach a threshold, and further anabolic pressure will not be regulated. Once this threshold is reached, adipocytes will be under pressure, and one of the responses to this pressure is to initiate the inflammatory process.

It is generally considered that the initial event of the inflammatory process is the imbalance of adipokine secretion from adipocytes, characterized by the reduced secretion of adiponectin and increased secretion of inflammatory mediators, including IL-6, leukotriene B4, and C-C motif chemokine ligand 2 (CCL2), which mediate circulating monocyte trafficking into the adipose tissue (59, 64, 65). Infiltrating monocytes and adipose tissue macrophages (ATMs) polarize into the pro-inflammatory subtype and attract additional monocytes by secreting their chemotactic and pro-inflammatory mediators (e.g., TNF-α, IL-1, and IL-6). The adipose tissue becomes inflamed, leading to chronic low-grade systemic inflammation (64, 66). Although the above process has been well-described, the cells that initiate the inflammatory cascade remain elusive. Several studies have shown that shifts in T cell composition in adipose tissue occur before monocyte-macrophage recruitment in obesity (67, 68). In lean mice, fat regulatory T (Tregs) and T helper type 2 (T_H_2) cells predominate in the adipose tissue and secrete IL-10 to block adipose tissue inflammation. There is a conversion in fat T cells during the onset of obesity, before the influx of macrophages allows CD8^+^ and T_H_1 adipose tissue T cells to dominate, and T_reg_ and T_H_2 cells are depleted. This transformation may initiate the trafficking of macrophages to fat *via* chemokines such as CCL2, which promotes local and systemic inflammation.

In obesity, the degree of inflammation in the adipose tissue varies in different anatomic regions. The subcutaneous adipose tissue is the largest lipid reservoir that prevents fat accumulation in organs under physiological conditions (69). Since the subcutaneous adipose tissue cannot store excess fat, overloaded fat accumulates, particularly in the visceral adipose tissue (70). Thus, visceral adipose tissue is a prominent site that becomes dysfunctional and inflamed during obesity, and it plays a significant role in cardiac pathophysiology. Given its close anatomical proximity to the heart and shared vascular supply, epicardial adipose tissue (EAT) also plays an essential role in myocardial remodeling in obesity mainly through mechanical and metabolic effects (70, 71). Metabolically, since myocardial and EAT depend on the same microvascular system, EAT in patients with obesity secrete a variety of adipokines including TNF-α, MCP-1, IL-6, IL-1β, fibrinogen activator inhibitor-1 (PAI-1), which can act directly on myocardial tissue through paracrine and vascular secretion, producing a proinflammatory state that promotes cardiomyocyte stiffness and coronary endothelial dysfunction (72). Mechanically, the enlarged EAT volume in patients with obesity might result in pericardial constrain. Indeed, the right atrial pressure of obese HFpEF, the measurable pressure closest to the pericardial pressure, increased during rest and exercise, suggesting the existence of pericardial restraint in obese HFpEF (42). Pericardial constraint may also lead to enhanced ventricular interdependence, which manifests as a higher ratio of LV and RV filling pressure, increased pulmonary venous pressure, and a larger LV eccentricity index (42, 73).

### Central role of low-grade systemic inflammation in obese HFpEF

Previous studies have suggested that hemodynamic changes and insulin resistance are leading causes of HFpEF (42, 57). However, in the recently proposed HFpEF paradigm, inflammation is considered the main pathophysiological factor of obese HFpEF (8, 54, 74). In cross-sectional studies of patients with HFpEF, higher circulating levels of TNF-α and IL-6 have been found (53, 54). Multiple studies have also demonstrated that inflammatory biomarkers such as TNF-α, IL-6, and hs-CRP are strongly associated with HFpEF incidence (53, 54, 75). Recently, a proteomic analysis to explore the inflammation paradigm in patients with HFpEF quantified 248 unique circulating proteins and showed that systemic inflammatory mediators were associated with echocardiographic indicators of worse hemodynamics and RV dysfunction. TNF-R1, UPAR, IGFBP-7, and GDF-15 were the top molecules that influenced the relationship between echocardiographic parameters and comorbidity burden (8). The obesity-induced systemic inflammatory status is implicated in several crucial processes in cardiac remodeling, including coronary microvascular and lymph-vessel dysfunction, cardiomyocyte hypertrophy and apoptosis, myocardial fibrosis, and consequently, HFpEF.

Coronary microcirculation disturbances are caused by the imbalance between endothelial-derived relaxing factors such as nitric oxide (NO) and contractile factors such as endothelin 1 (76). Pro-inflammatory cytokines are known to trigger the overproduction of endothelial reactive oxygen species (ROS) by activating nicotinamide adenine dinucleotide phosphate (NADPH) oxidase (NOX) (77), which induces increased expression of high nitrotyrosine and reduces NO bioavailability. Studies have found that plasma NO metabolite levels are lower in patients with HFpEF than in patients with HFrEF, confirming the reduced NO bioavailability in patients with HFpEF (78). Consequently, reduced NO bioavailability results in impaired NO-cyclic guanosine monophosphate-protein kinase G (NO-cGMP-PKG) signal transduction pathway, thereby causing the hypertrophy and stiffening of surrounding cardiomyocytes (54, 79). Along with the altered paracrine signaling between microvascular endothelium and cardiomyocytes, reduced myocardial microvascular density, known as rarefaction, is observed in patients with HFpEF. This impairs cardiac perfusion and myocardial oxygen delivery, leading to a deteriorated diastolic function in patients with HFpEF (24, 25).

Coronary microvascular endothelial dysfunction drives cardiomyocyte stiffening and hypertrophy by lowering myocardial NO bioavailability and PKG activity, which are critical mechanisms in obese HFpEF (54). In addition, robust evidence suggests that sustained stimulation of inflammatory cytokines has a direct detrimental impact on the heart (80). At the cellular level, inflammatory cytokines can directly bind to cardiomyocyte surface receptors, triggering the downstream signaling of cellular inflammation, hypertrophy, and apoptosis (80–82). TNF-α, for example, initiates the signaling cascade involved in cardiomyocyte hypertrophy from the cell membrane by binding to two different TNF receptors (83, 84). Moreover, IL-1β affects the progression of cardiomyocyte hypertrophy through insulin-like growth factor-1 (IGF-1) production, which negatively regulates c-Jun N-terminal kinase (JNK) signals (85). Consistently with the *in vitro* results, IL-1 receptor type I-deficient mice were protected from aging-exacerbated HFpEF, due to being unresponsive to IL-1 (86).

The extensive lymphatic network of the heart is critical for maintaining cardiac fluid balance, in which the lymphatic reflux is mainly driven by cardiac contraction (87). Lymphatic dysfunction and remodeling have been observed in various experimental models of HFpEF-related comorbidities (88, 89). Various immune cells were attracted to the lymphatic vessels, reducing the pumping rate of dermal lymphatic collection vessels and decreasing the density of lymphatic capillaries. This leads to reduced lymphatic transport in several preclinical obesity models (90, 91), implying a role of inflammation in lymphatic dysfunction.

The accumulation of excessive extracellular matrix and cardiac fibrosis are hallmarks of deleterious remodeling that occurs during HFpEF (24, 92, 93). Pro-inflammatory cytokine IL-16 and macrophage infiltration in the myocardium reportedly contribute to increased cardiac fibrosis and LV stiffness in HFpEF (94, 95). Furthermore, microvascular rarefaction is also associated with myocardial fibrosis in patients with HFpEF, suggesting that decreased cardiac perfusion may cause overproduction and accumulation of collagen (24). Interestingly, cardiac edema and inadequate lymphatic transport increase collagen production. In contrast, the reduction of cardiac edema by accelerating cardiac lymphangiogenesis alleviated interstitial fibrosis in a rodent model of myocardial infarction, implying a possible role of inflammation in regulating cardiac interstitial fibrosis through edema and poor lymphatic transport (96).

### Direct pro-inflammatory effects of nutrients overload on theheart: The central role of cardiac metabolic reprogramming

In addition to circulating inflammatory factors, nutrient overload has recently been found to directly induce cardiac inflammation mainly through cellular metabolic reprogramming, particularly in myocytes (59). In normal hearts, over 95% of the energy is produced by mitochondrial oxidative phosphorylation. Free fatty acids (FFAs) are the primary fuel substrates for the myocardium, with the remaining 30% coming from glucose and other metabolites such as lactate (97). Normally, the heart exhibits remarkable fuel flexibility and switches between glucose and fatty acids, depending on the energy requirements of the heart and the body’s nutritional status. The glucose-fatty acid cycle of Randle controls the coordinated regulation of metabolic substrate utilization, which is important for the maintenance of the heart’s energy homeostasis (98).

In obesity, elevated FFA levels upregulate the expression of genes involved in fatty acid oxidation (FAO) in cardiomyocytes by activating the nuclear receptor peroxisome proliferator-activated receptor α. Once the enhanced FFA uptake exceeds the FAO capacity, excess FFAs are stored as triglycerides, which mediate the ectopic triglyceride accumulation in the heart and lipotoxicity (99). Increased FFA utilization and lipotoxicity favor ROS overproduction, and in turn, activate distinct cellular inflammatory signaling cascades, resulting in myocyte hypertrophy, apoptosis, and cardiac insufficiency (100). Furthermore, increased FAO inhibits insulin-dependent glucose transporter 4 (GLUT4)-mediated glucose uptake and utilization, which is associated with insulin resistance, thereby leading to the exposure of cardiomyocytes to high glucose levels. Hyperglycemia can directly induce a series of pro-inflammatory pathways in cardiomyocytes that converge toward NF-κB, including the activation of mitogen-activated protein kinase and JNK, which promotes the upregulation of TNF-α, IL-1β, IL-6, and IL-12 (101). In addition, the upregulation of histone H3 lysine 9 trimethyl at the IL-6 promoter with high-glucose stimulation also favors cardiomyocyte inflammation (102). Recently, a novel molecular pathway by which hyperglycemia triggers direct O-GlcNAcylation of Ser280 in Ca^2+^/calmodulin-dependent kinase II and the consequent activation of NOX was identified in cardiomyocytes (103). This leads to increased cytosolic ROS accumulation and the subsequent activation of cellular inflammation (103, 104).

In addition to cardiomyocytes, nutrient overload can directly induce inflammation in other types of non-cardiomyocytes, such as macrophages and endothelial cells. Resident cardiac macrophages play essential roles in maintaining myocardial equilibrium during steady-state and tissue repair in the injured heart. At a steady state, cardiac macrophages resemble alternatively activated anti-inflammatory macrophages and function in homeostasis by helping to remove senescent dead cells and defending against infection without inducing an immune response (105). These alternatively activated macrophages primarily undergo FAO and oxidative phosphorylation to produce ATP. In response to pro-inflammatory stimuli, macrophages reprogram their metabolism to rapid aerobic glycolysis because of the increased demand for energy (106).

In contrast to cardiomyocytes, macrophages are naturally insulin-resistant and take up glucose through insulin-independent GLUT1. Thus, in obesity-related insulin resistance, macrophages can sense glucose availability and dynamically reprogram their core metabolism by shifting toward cytosolic glycolysis and away from mitochondrial respiration, polarizing themselves away from the anti-inflammatory subtype and toward the pro-inflammatory subtype through glycolysis intermediates. For example, lactate has been shown to increase histone lactylation to induce homeostatic gene expression in macrophages (107). Moreover, glycolysis supplies metabolic substrates, such as glucose-6-phosphate, for the pentose phosphate pathway (PPP) to generate NADPH, which can be used by NOX to produce ROS and support the synthesis of inflammatory leukotrienes and prostaglandins, thereby triggering NF-kB signaling (59, 108). Enhanced glycolysis leads to suppression of the pyruvate funnel into the mitochondria, followed by a broken tricarboxylic acid cycle that results in succinate accumulation, which boosts hypoxia-inducible factor-1α (HIF-1α) activation of pro-inflammatory IL-1β gene expression (109).

The preference for substrate utilization in endothelial cells, particularly arterial endothelial cells, differs from that of cardiomyocytes and macrophages. Because of its low energy demand, glycolysis is the predominant glucose utilization pathway, whereas FFAs are not an important fuel in coronary endothelial cells in the normal state (110, 111). Recently, Wang et al. (112) demonstrated that metabolism in endothelial cells is reprogrammed during inflammatory stimuli, manifested as activated glycolysis, PPP, and mitochondrial respiration. While blocking the metabolic enhancement of glycolysis attenuates inflammation, inhibiting the activated PPP and mitochondrial respiration causes an aggravation of inflammation, implying the role of activated PPP and mitochondrial respiration as homeostatic anti-inflammatory mechanisms, which is different from that in macrophages.

Altogether, it is clear that the preference of different cells for metabolic substrates leads to different metabolic phenotypes in obese HFpEF. This phenomenon is important and should be considered in therapies targeting myocyte metabolism with unknown effects on other cardiac cells that continue to be prescribed.

## NLR family pyrin domain containing 3 (NLRP3) inflammasome activation: A key culprit?

**Figure 3:**
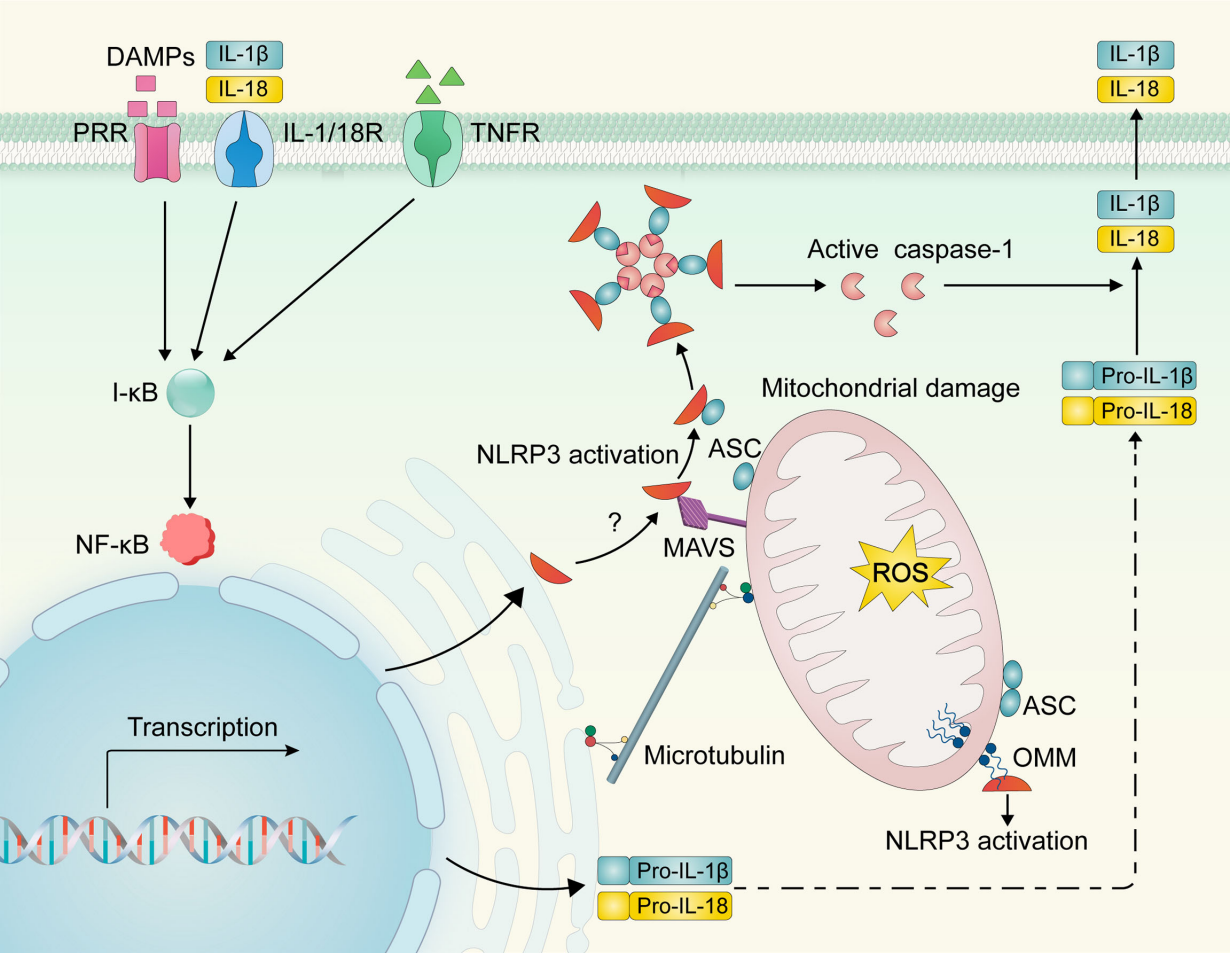
NLRP3 inflammasome activation. DAMPs and cytokines (e.g., IL-1β/18 and TNF-α) can bind to PRR and cytokine receptors, respectively, to activate the I-κB/NF-κB signaling pathway and induce NLRP3 and pro-IL-1β/IL-18 transcriptional upregulation. Upon activation, NLRP3 may be recruited from the ER to the mitochondria and interact with mitochondrial molecules (e.g., mitochondrial antiviral signaling protein and cardiolipin). Dynein-mediated mitochondria may regulate the interaction of NLRP3 and ASC *via* microtubule transport, resulting in inflammasome assembly, caspase-1 activation, and subsequent activation of IL-1β and IL-18. *NLRP3—NOD-like receptor family, pyrin domain containing 3; DAMPs, damage-associated molecular patterns; PRR, pattern recognition receptor; IL-1/18R, interleukin-1/18 receptor; TNFR, Tumor necrosis factor receptor; NF-κB, nuclear factor kappa B; I-κB, inhibitory protein of NF-κB; pro-IL-1β/18, pro-interleukin-1β/18; ER, endoplasmic reticulum; OMM, outer mitochondrial membrane; ROS—reactive oxygen species; MAMs, mitochondria associate membranes; MAVS, mitochondrial antiviral signaling protein; CL, cardiolipin; ASC, apoptosis-associated speck-like protein containing a caspase recruitment domain*.

The innate immune system, which is the first line of defense of the host immune system, detects pathogens or host tissue damages, also known as pathogen-associated molecular patterns or damage-associated molecular patterns (DAMPs), respectively, mediated by pattern recognition receptors (PRRs). Upon recognition of DAMPs, some PRRs, including Nod-like receptors (NLRs) and Toll-like receptors (TLR), can assemble with other proteins to form multi-molecular complexes termed inflammasomes. Currently, the NLRP3 inflammasome is the most well-characterized inflammasome and is composed of the NLRP3, the adaptor protein ASC (apoptosis-associated speck-like protein containing a caspase recruitment domain), and pro-caspase-1 (113). The canonical NLRP3 inflammasome activation usually requires two steps: initial priming and subsequent inflammasome assembly (**Figure 3**). The first priming signal involves PRR or cytokine receptor activation to upregulate NLRP3 and pro–IL-1β/IL-18 transcription. NLRP3-specific stimulus (e.g., mitochondrial ROS, potassium efflux, long-chain saturated FAs, ceramide, and glucose) is implicated in NLRP3 activation (114, 115). Upon triggering, NLRP3 recruits the adapter molecule ASC to catalyze pro-caspase-1 maturation. Activated caspase-1 in turn cleaves pro-IL-1β/IL-18 and induces the release of their mature form (60, 116). IL-1 can induce the production and release of copious amounts of IL-6 from many cell types in an amplification loop (117).

Resting NLRP3 localizes to endoplasmic reticulum (ER) structures. However, a point of confusion is where the NLRP3 assembly occurs. Mitochondrial damage and ROS have been shown to act as upstream mechanisms of NLRP3 activation, implying a role of the mitochondria in NLRP3 inflammasome assembly. An informatics-based structure-function analysis predicted that NLRP3 localizes to mitochondria (118). Subsequently, several studies have shown that during NLRP3 inflammasome activation or overexpression, NLRP3 is recruited to the mitochondria and mitochondria-associated membranes, which are contact sites for the mitochondrial membrane and ER (119, 120). This recruitment requires a mitochondrial antiviral signaling protein whose interaction with NLRP3 depends on the N-terminal sequence of the latter molecule (121). Cardiolipin translocates from the inner mitochondrial membrane to the outer mitochondrial membrane and provides a docking site for NLRP3 in the impaired mitochondria (122).

There has been increasing recognition that NLRP3 inflammasome activation acts as a chief instigator of obesity, contributing to obesity-related systemic inflammation and insulin resistance. A Study by Stienstra et al. (123) found that the NLRP3 inflammasome contributes to the development of obesity and its comorbidities during high-fat diet (HFD) feeding in mice. Ablation of NLRP3 and deficiency of the inflammasome component, ASC or caspase-1, represses the inflammatory cascade response of inflamed adipose tissue and improves insulin signaling (123, 124). Mechanistically, it has been proposed that lipid messengers (e.g., palmitate and ceramides), which are elevated in both individuals with obesity and HFD-challenged mice, act as signals for NLRP3 inflammasome priming and activation (125–129). Previous studies have recognized that the elevation of palmitate in the context of obesity may induce NLRP3 inflammasome activation in macrophages, leading to the overproduction of inflammatory cytokines and subsequent insulin resistance in mice (125, 126). Vandanmagsar et al. (116) showed that the NLRP3 inflammasome senses ceramide in order to induce caspase-1 cleavage in macrophages and adipose tissue, thereby contributing to obesity-related inflammation and comorbidities.

In addition to the production of systemic inflammation originating from inflamed adipose tissue, recent research has shown that activation of the NLRP3 inflammasome is closely related to the severity of the HFpEF phenotype. Mice with HFpEF show NLRP3 inflammasome activation and overproduction of cytokines IL-1β/IL-18 in the heart (60, 130). Cardiac muscle mitochondrial abnormalities and ROS overproduction, which are important factors contributing to the pathophysiology of HFpEF, play a key role in activating the NLRP3 inflammasome (131, 132). Mitochondrial protein hyperacetylation in obese HFpEF increases the amount of ASC in mitochondria, exacerbating inflammation by facilitating NLPR3 inflammasome assembly in mitochondria and subsequent release of IL-1β/IL-18 and cardiac fibrosis (60). Collectively, these results could point to a key role of NLRP3 inflammasome in the pathophysiology of obese HFpEF and indicate that it should be inhibited to improve prognosis.

### Targeting drug therapy of HFpEF: the road ahead and an exciting future

NLRP3 inflammasome, which is an important mediator in the development of obese HFpEF, is regarded as a promising therapeutic target due to its important role. This section focuses on drugs that directly or indirectly suppress inflammasome assembly and activation.

### IL-1R/IL-1 inhibitors

Therapeutic targeting of the NLRP3 inflammasome in patients with HF has commonly used the IL-1 receptor antagonist (IL-1Ra) anakinra, thereby inhibiting signaling of both IL-1α and IL-1β. The D-HART pilot trial in patients with HFpEF showed that short-term IL-1 blockage with anakinra led to a significant reduction in systemic inflammatory mediators and improvement in peak oxygen consumption (V_O2_) and aerobic exercise capacity (133). Subsequently, the D-HART2 trial, in which 12 weeks of treatment resulted in a significant decline in NTpro-BNP concentrations compared with placebo, further confirmed the benefits of anakinra in the treatment of HFpEF (134).

Although these two trials suggested that blocking IL-1 is a promising treatment strategy for HFpEF with systemic inflammation, it also raises concerns regarding its safety because it interferes with immune homeostasis. Canakinumab, a monoclonal antibody that specifically neutralizes IL-1β and does not affect other IL-1 family cytokines, may be safer than anakinra. Although no clinical trials with canakinumab included patients with HFpEF, in a small substudy of the Canakinumab Anti-Inflammatory Thrombosis Outcome Study (CANTOS) trial, 150 mg canakinumab treatment improved peak V_O2_ and increased LVEF in patients with HFrEF, implying the potential of canakinumab in HF treatment (135).

In addition to IL-1, IL-6 blockade in mice through genetic deletion is associated with decreased cardiac hypertrophy and fibrosis following angiotensin II stimulation (136). Although no clinical studies have explored anti-IL-6 therapeutics in patients with HFpEF, the findings regarding IL-6 and HFpEF development in the preclinical model warrant further exploration to investigate whether IL-6 is an appropriate therapeutic target.

### Colchicine

Colchicine, the major alkaloid in *Colchicum autumnale*, is a potent drug with anti-inflammatory properties. It acts on inflammation through different mechanisms, with the inhibition of spindle microtubule assembly *via* copolymer formation being the best characterized (137). Recently, colchicine has been identified as a non-specific IL-1β inhibitor that blocks the activation of the cellular NLRP3 inflammasome, thus reducing the release of pro-inflammatory IL-1β/18 (138–143). Although colchicine is currently recommended for the treatment of acute gout attacks and familial Mediterranean fever, it has historically demonstrated benefits in various cardiovascular diseases, including postpericardiotomy syndrome, acute and recurrent pericarditis, postoperative atrial fibrillation, and chronic HF (144). In addition, clinical evidence of its action in coronary artery disease (CAD) has yielded encouraging results.

The low-dose colchicine (LoDoCo) pilot study and LoDoCo2 trial conducted by Nidorf et al. (145, 146) established a significant benefit of low-dose colchicine in preventing major adverse cardiovascular events in patients with stable CAD. Subsequently, the large-scale Colchicine Cardiovascular Outcomes Trial (COLCOT) tested low-dose colchicine in patients who experienced a recent myocardial infarction (MI) and served as a strong validation for the potential benefits of low-dose

colchicine in acute coronary syndrome (147). In addition to its effectiveness, the use of colchicine at 0.5–1.0 mg daily in cardiovascular trials has been proven safe in the absence of severe liver or renal disease. The most common side effects are gastrointestinal problems, which limit its usefulness in approximately 10% of patients; however, approximately 90% of patients tolerate the long-term continuous treatment well (148).

The effectiveness of low-dose colchicine in atherosclerotic cardiovascular disease, a chronic inflammatory disease, raises the question of whether inhibiting NLRP3 inflammasome-related inflammation with colchicine is effective in the treatment of obese HFpEF. Currently, evidence that colchicine can reduce the risk of HF is limited to a single randomized controlled trial that showed colchicine reduced the circulating inflammatory biomarker levels and had favorable effects on LV remodeling in patients with HFrEF. However, this did not translate into an impact on clinical outcomes, exercise capacity, or improvements in mortality (149). Considering the importance of chronic inflammation in the pathogenesis of obese HFpEF, the effectiveness of colchicine in HFpEF is worth exploring.

### β-hydroxybutyrate

β-hydroxybutyrate (BHB) is a ketone metabolite tested by Youm et al. (150) for NLRP3 inflammasome blockade. Mechanistically, BHB inhibits the activation of the NLRP3 inflammasome by preventing the efflux of potassium and reducing ASC oligomerization and speck formation. Recently, in an HFpEF mouse model, by combining age, long-term HFD, and desoxycorticosterone pivalate challenge, Deng Y et al. (60) further proved that increasing the abundance of BHB blocked NLPR3 inflammasome formation and alleviated pro-inflammatory IL-1β/18-triggered mitochondrial dysfunction and myocardial fibrosis. These findings suggest that dietary or pharmacological attempts to increase BHB levels may be effective treatments for HFpEF. Caloric restriction, which increases BHB abundance, prevents inflammatory processes, thereby improving myocardial remodeling and LV diastolic function in both preclinical models and humans (23, 151, 152).

Sodium-dependent glucose co-transporter 2 (SGLT-2) inhibitors, which have been found to increase systemic and tissue BHB levels, thereby attenuating NLRP3 inflammasome activation and the development of HFpEF (153, 154), are another promising intervention for patients with obese HFpEF. In a multicenter randomized trial that evaluated the effect of SGLT2 inhibitor dapagliflozin on the Kansas City Cardiomyopathy Questionnaire Clinical Summary Score (KCCQ-CS) (155) in HFpEF patients, found that after 12 weeks of treatment with dapagliflozin, the KCCQ total symptom score, physical restriction score, and 6-minute walk test were improved, suggesting that SGLT2 inhibitor treatment improved the symptoms, physical restriction and motor function of patients with HFpEF. The recently concluded DELIVER trial which investigated the effects of dapagliflozin on major adverse cardiovascular events in patients with HFpEF, and reached a statistically significant and clinically meaningful reduction in the primary composite endpoint of cardiovascular CV death or worsening HF (156). In addition to dapagliflozin, the recently published EMPEROR-Preserved clinical trial explored the effect of empagliflozin (another SGLT2 inhibitor) on the primary composite endpoint of HF hospitalization or cardiovascular death in patients with HF and an EF of >40% (157). Empagliflozin produced a significant, early, and sustained reduction in the combined risk, regardless of the presence or absence of diabetes. These trials definitively defined the role of SGLT-2 inhibitors in patients with HFpEF (158).

### The challenges from HFpEF animal models

As previously mentioned, although some therapeutic agents that directly or indirectly inhibit the NLRP3-IL-1/IL-6 pathway could turn out to be promising strategies against obese HFpEF, some have intolerable side effects and still need clinical trials. Future studies should focus on the discovery of drugs with specific targets and improved pharmacokinetic properties. Valuable HFpEF animal models that mimic the clinically obese HFpEF phenotype are essential for the successful development of novel drugs. Currently, the lack of suitable preclinical obese HFpEF models that adequately reflect the disease’s clinical complexity has led to the inability to determine the mechanistic models and develop new therapeutic agents.

The development of obese HFpEF in humans is the result of multiple comorbidities, including obesity, prediabetes, and pulmonary and systemic arterial hypertension, which are difficult to reproduce in one animal model. Recently, two diagnostic algorithms (H2FPEF and HFA-PEFF scores) have been proposed to standardize the diagnosis of human HFpEF (3, 159). Withaar et al. (160) evaluated the translational value of HFpEF mouse models in the context of these two clinical scores and found that most HFpEF animal models do not meet the clinical diagnostic criteria for HFpEF, although some multi-hit models simulate human HFpEF to a reasonable extent. Several typical obese HFpEF animal models and their pathophysiological characteristics are shown in **Table 1**.

**Table 1:** Pathophysiology characteristics of Obese HFpEF animal models.

Model	Comorbidities				HFpEF Pathophysiology					
	Obesity	HC	(pre-) DM	HT	LV Structure	Systolic Function	Diastolic dysfunction	Pulmonary Congestion	Exercise Intolerance	Limitation
**One-hit pre-clinical model**										
**db/db mouse**(161)	Yes	Yes	Yes	No	Concentric hypertrophy	Preserved	Yes	Yes	Yes	Confounding effects of leptin-mediated signaling changes
**ob/ob mouse**(162)	Yes	Yes	Yes	No	Concentric hypertrophy	Preserved	Yes	Not described	+/-	Confounding effects of leptin-mediated signaling changes
**ZSF1 rat**(163)	Yes	Not described	Yes	Yes	Concentric hypertrophy	Preserved	Yes	Yes	Yes	Additional comorbidities confound the findings
**High-fat diet/western-diet mouse**(164)	Yes	Not described	Yes	Yes	Concentric hypertrophy	+/-	Yes	No	yes	Different total fat contents and durations according to these dietary treatments
**Cholesterol-enriched diet New Zealand white rabbits**(165)	Yes	Yes	Yes	Not described	Hypertrophy	Preserved	Yes	Not described	Not described	Different total fat contents and durations according to these dietary treatments
**Two-hit pre-clinical model**										
**db/db and Ang II mouse**(166)	Yes	Yes	Yes	Yes	Concentric hypertrophy	Preserved	Yes	Yes	Not described	Confounding effects of leptin-mediated signaling changes
**High-fat diet and STZ mouse**(167)	Yes	Not described	Yes	No	Eccentric hypertrophy	Reduced	Yes	Not described	Not described	Time-dependent progression of the phenotype
**High-fat diet and STZ minipigs**(168)	Yes	Yes	Yes	Not described	Not described	Not described	Yes	Not described	Not described	High housing cost and time-consuming
**High-fat diet and L-NAME mouse**(169)	Yes	Not described	Yes	Yes	Concentric hypertrophy	Preserved	Yes	Yes	Yes	Sex-dependent effects
**High-fat diet and Ang II mouse**(170)	Yes	Not described	Yes	Yes	Concentric hypertrophy	Preserved	Yes	No	Not described	Clinical features are mild
**Multi-hit pre-clinical model**										
**High-fat diet, aging, and Ang II infusion**(171)	Yes	Not described	Yes	Yes	Concentric hypertrophy	Preserved	Yes	Yes	Not described	Time-consuming
**High-fat diet, aging, and DOCP mouse**(60)	Yes	Not described	Yes	Yes	Hypertrophy	Preserved	Yes	Yes	Yes	Time-consuming
**High-fat diet, aging, female sex, and Ang II infusion mouse**(171)	Yes	Not described	Yes	Yes	Concentric hypertrophy	Preserved	Yes	Yes	Yes	Potential sex-specific effects
**High-fat fructose, salt-diet, and excess mineralocorticoid minipigs**(172)	Yes	Yes	Yes	Yes	Concentric hypertrophy	Preserved	Yes	Yes	Not described	High housing costs and time-consuming

HFpEF Pathophysiology, pathophysiology of heart failure with preserved ejection fraction; HC, hypercholesterolemia; (pre-) DM, pre-stage of diabetes mellitus; HT, hypertension; LV Structure, Left ventricular structure; db/db mouse, leptin receptor-deficient mouse mode; ob/ob mouse, leptin-deficient mouse model; ZSF1 rat, the diabetic Zucker fatty spontaneously hypertensive heart failure F1 hybrid rat model; High-fat diet/western-diet mouse, mouse model fed with high-fat diet or western-diet; Cholesterol-enriched diet New Zealand white rabbits, New Zealand white rabbits model fed with the diet of rich in cholesterol; db/db and Ang II Mouse, leptin receptor-deficient mouse model perfused with angiotensin II; High-fat diet and STZ mouse, mouse model injected with streptozotocin and fed with high-fat diet; High-fat diet and STZ minipigs, minipigs model injected with streptozotocin and fed with high-fat diet; High-fat diet and L-NAME mouse; mouse model injected N(w)-Nitro-L-Arginine Methyl Ester Hydrochloride(constitutive nitric oxide synthase inhibitor);and fed with high-fat diet; High-fat diet and Ang II mouse, mouse model fed with high-fat diet and perfused with angiotensin II; High-fat diet, aging, and Ang II infusion, mouse models treated with ageing,high-fat diet and angiotensin II; High-fat diet, aging, and DOCP mouse, mouse models treated with aging,high-fat diet and desoxycorticosterone pivalate; High-fat diet, aging, female sex, and Ang II infusion mouse, mouse models treated with aging, female sex, high-fat diet and angiotensin II; High-fat fructose, salt-diet, and excess mineralocorticoid minipigs, minipigs models treated with excess mineralocorticoid and high-fat fructose and salt-diet.

Several methods can be used to induce obesity in laboratory animals, including the use of genetically modified mice. Obese mouse models lacking the leptin gene (ob/ob) and leptin receptor (db/db) show signs of HFpEF, including concentric hypertrophy, exercise intolerance, and pulmonary hypertension (173). Obesity is characterized by leptin resistance in humans; however, the mouse model relies on leptin deficiency and does not accurately recapitulate the clinical obese HFpEF phenotype (174, 175). Similarly, the obese ZSF1 (Zucker fatty spontaneously hypertensive heart failure F1 hybrid) rat, which was developed by crossing rat strains with two separate leptin receptor mutations, also exhibited signs of HFpEF (elevated LVEDP, elevated E/e, and preserved LVEF) (163, 176). Notably, in addition to obesity and metabolic abnormalities, the ZSF1 model presents other comorbidities, such as spontaneous time-dependent chronic kidney disease or hypertension, which may be considered to mimic human HFpEF in many scenarios. However, if the aim is to discern the contribution of obesity to HFpEF, then the additional chronic kidney disease would confound the findings (173).

To avoid potential perturbations based on altered leptin expression and signaling in genetically modified models, numerous dietary treatment regimens have been used to induce obesity in rodents, which has become a standard approach in preclinical studies. HFD usually contains a total fat content of up to 60%, whereas a Western diet typically has a high-fat content and commonly uses sucrose. Cardiac abnormalities in the diet-induced obesity model vary depending on the total fat content and the duration of these dietary treatments, thereby limiting pre-clinical utility (177). For example, mice challenged with HFD with 60% fat content developed systolic dysfunction after 10 weeks of feeding and had an increase in mortality (178). In contrast, exposure to a Western diet leads solely to diastolic dysfunction, while preserving systolic function (164). To overcome the potential low penetrance of diabetes development in diet-induced obesity, several studies have used HFD plus low-dose streptozotocin to induce β-cell dysfunction and diabetes. Additional treatment with streptozotocin in HFD-challenged mice promoted eccentric cardiac hypertrophy and LV diastolic dysfunction (167, 179, 180).

Given that the development of obese HFpEF in humans is the result of several comorbidities, combinatory models of HFD and other factors have been evaluated. Schiattarella et al. (169) combined HFD and hypertension induced by inhibition of nitric oxide synthase to produce a 2-hit preclinical mouse model that resembles human obese HFpEF. However, sex-dependent effects, which are an obstacle to preclinical application, have been reported (181). The combination of HFD and angiotensin II (Ang II)-dependent hypertension also resulted in HFpEF. However, symptoms, signs, and hemodynamic features appear to be mild, since the effect on exercise capacity is unknown and lung congestion in young animals is absent. Recently, Deng et al. (60) developed a 3-Hit strategy consisting of a 13-month HFD and 16 months of aging, followed by an intraperitoneal injection of a bolus of desoxycorticosterone pivalate for 1 month. By the end of the 13 months, 3-hit-treated mice manifested pathological myocardial hypertrophy and fibrosis, diastolic dysfunction, as well as pulmonary congestion, and elevated natriuretic peptides, which recapitulated the typical pathological phenotype of obese HFpEF. However, generating this model is time-consuming and not conducive to testing potential new therapeutic agents. Because female sex was independently associated with the presence of diastolic dysfunction and worse clinical outcomes in HFpEF patients (182), Withaar et al. (171) developed a multi-hit model which combined advanced age, female sex, and an HFD with Ang II infusion. Although this HFpEF model doesn’t represent the entire spectrum of human HFpEF, it provides a sex-specific approach for exploring the pathophysiology of HFpEF.

In addition to the limitations mentioned above, the hearts of rodent models are not comparable in size, structure, and function to the hearts of humans. Therefore, there is an urgent need for large-animal HFpEF models that can recapitulate the complex features of obese HFpEF in humans. The currently available methods for generating large animal models of HFpEF are summarized in detail elsewhere (174, 175). A recent study proposed an HFpEF model in Göttingen minipigs, which induced hypertension and obesity through mineralocorticoid excess and high cholesterol, fat, fructose, and salt diet (172). This novel “multi-hit” minipig model of HF is free from the confounding effects inherent in hypertension and end-organ damage induced by invasive surgical procedures or artificial drug administration and can be a promising model used for molecular mechanisms and evaluation of potential therapeutic approaches to HFpEF.

*In vitro* cardiomyocyte models of humans can overcome the limitations of animal models and species differences. However, *in vitro* studies of cardiomyocytes have many technical challenges. Current *in vitro* cardiomyocyte models that can be used to simulate HFpEF include induced pluripotent stem cells (iPSCs), primary cells, and cardiac organoid models (174). The iPSC-derived cardiomyocytes (iPSC-CMs) usually dedifferentiate from fibroblasts or monocytes and differentiate into cardiomyocytes under different *in vitro* epigenetic and environmental regulations, which are unable to produce a mature phenotype that is representative of human adult ventricular cardiomyocytes. Primary cell culture can obtain pathological changes in cardiomyocytes from HFpEF patients, but it is difficult to obtain and the cells rapidly lose their phenotypic structure in a relatively short period after extraction (183). The cardiac organoid model can overcome the limitations of single-molecule culture. However, this model is still artificially constructed and the resulting phenotype is very different from the intact human myocardium (184).

Recently, a new human functional myocardial slicing method was proposed, in which isolated myocardial tissue was prepared into ultrathin slices (150–350 μm) of ventricular myocardium and cultured under mechanical tension and electric field stimulation (174). This method preserves the structure of the failing myocardium and its molecular characteristics and can be used to study the function of the failing myocardium, energy-substrate turnover, and molecular properties, as well as the cardiac response to drug treatment.

In summary, based on the advantages and disadvantages of each model, we advocate that future HFpEF studies that explore molecular mechanisms and screen for potential new drugs should consider the use of multiple HFpEF animal models to narrow the gaps in HFpEF pathophysiology and promote the study of molecular mechanisms and the development of HFpEF therapeutics.

## Conclusion

Obesity is an independent risk factor for myocardial dysfunction and HF. Obese HFpEF has a unique cardiac remodeling pattern and pericardial restraint mechanics. Inflammation is considered the main pathophysiological factor in obese HFpEF. We propose inflammation-related mechanisms of obese HFpEF, as well as potential therapeutic approaches. Currently, treatment options for HFpEF are limited, and the pharmacological inhibition of NLRP3 inflammasome assembly and activation may provide a novel treatment strategy targeting obese HFpEF. However, the discovery of novel therapeutics is hindered by the lack of appropriate HFpEF animal models that recapitulate this complex, comorbidity-laden, heterogeneous disease. Successfully validated large animal models or *in vitro* models of human cardiomyocytes could provide a more comprehensive understanding of the complex paradigm of HFpEF and potentially serve as reliable preclinical platforms for testing its therapeutic potential.

## Author contributions

YB directed the writing and revision of the thesis. CL and DQ wrote the article and generated figures and tables. DH, JH and YY contributed to the mechanisms of the paper. All authors contributed to the article and approved the submitted version.

## Funding

This work was supported in whole or in part by the National Natural Science Foundation of China (No.82170483 to BY and No.82100496 to DH) and the Natural Science Foundation of Hunan Province of China (No.2020JJ4786 to JH).

## Acknowledgments

All authors are grateful to Zhenyu Tian for illustrating our manuscript.

## Conflict of interest

The authors declare that the research was conducted in the absence of any commercial or financial relationships that could be construed as a potential conflict of interest.

## Publisher’s note

All claims expressed in this article are solely those of the authors and do not necessarily represent those of their affiliated organizations, or those of the publisher, the editors and the reviewers. Any product that may be evaluated in this article, or claim that may be made by its manufacturer, is not guaranteed or endorsed by the publisher.
